# A New Corpus of Lexical Substitution and Word Blend Errors: Probing the Semantic Structure of Lemma Access Failures

**DOI:** 10.5334/joc.278

**Published:** 2023-05-18

**Authors:** John Alderete, Melissa Baese-Berk, Adrian Brasoveanu, Jess H. K. Law

**Affiliations:** 1 Linguistics, Cognitive Science, Simon Fraser University, Burnaby, V5A 1S6, Canada; 2 University of Oregon, Eugene, United States; 3 Linguistics, University of California Santa Cruz, Santa Cruz, United States

**Keywords:** lemma selection, speech errors, lexical substitutions, word blends, semantic processing

## Abstract

Models of lemma access in language production predict occasional mis-selection of lemmas linked to highly similar concepts (synonyms) and concepts standing in a set-superset relation (subsumatives). It is unclear, however, if such errors occur in spontaneous speech, and if they do, whether humans can detect them given their minimal impact on sentence meaning. This data report examines a large corpus of English spontaneous speech errors and documents a low but non-negligible occurrence of these categories. The existence of synonym and subsumative errors is documented in a larger open access data set that supports a range of new investigations of the semantic structure of lexical substitution and word blend speech errors.

## 1. Introduction

Speech errors have long been used as a lens on language production, providing empirical support for models of lexical access, grammatical and form encoding, and articulatory processing (Bock & Levelt, 1994; Dell et al., 2021; Levelt et al., 1999; Rapp & Goldrick, 2000). Despite this progress, errors involving the mis-selection of lemmas (i.e., words with syntactic and semantic information but lacking phonological structure) remain elusive. Descriptively, we know that these errors either blend two words (e.g., /Papa, Dad/→*Pad*) or substitute one for another meaning-related word, as in /lunch/→*dinner*. Many questions remain, however, about the semantic relationships of these errors, the relative frequencies of different types, and how to predict their occurrence.

Levelt (1989) presented the first coherent analysis of lemma access errors by distinguishing intrusions at two levels of processing. Word blends, which are dominated by synonyms, involve mis-selections at the conceptual level between two highly confusable concepts. Lexical substitutions, on the other hand, tend to involve word associates, like antonyms and co-hyponyms, and arise through competition at the lemma level. Can conceptual intrusions (i.e., synonyms) propagate into the lemma level and surface as lexical substitutions? Though the data available at the time (Garrett, 1982; Hotopf, 1980) suggested no, Levelt (1989) asks an important empirical question that motivates our data report. Synonym errors, as well as super-ordinates (e.g., /dog/→*animal*), have broadly similar truth conditions, and so they may have little impact on sentence meaning. Since lexical substitutions leave no trace of the intended word (cf. word blends), how will data collectors even know that an error occurred?

This issue is important because current models of lemma access predict lexical substitutions with synonyms and subsumatives (i.e., super-ordinates and sub-ordinates). Though highly confusable synonyms are unique in conceptual processing, synonym and subsumative word pairs are nonetheless associated, so they are predicted at the lemma level as well. In WEAVER++ and swinging lexical network models, semantically related words that share a super-ordinate compete for selection because of activation spreads to and from the shared concept (Abdel Rahman & Melinger, 2009; Roelofs, 1992). If errors arise from this competition, we expect synonym and subsumative errors by the same mechanism. Finally, semantic errors in the two-step interactive model (Dell et al., 1997; Schwartz et al., 2006) arise from the activations of shared semantic features. Surely, synonym and subsumative pairs share as many or more semantic features as co-hyponyms and antonyms, so we expect errors with them as well.

Though lacking in early research, two studies have provided some positive evidence for the errors in question. Jaeger (2005) examined 215 lexical substitutions in adults and children and found a dozen errors with (near) synonyms and 15 subsumative errors. In this study, ‘near synonym’ errors involve words that refer to the essentially same entity but differ in selectional restrictions that constrain their use, as in /ripe avocado/→ *done avocado*. In a larger collection of Mandarin errors, Wan and Ting (2019) also documented (near) synonym and subsumative lexical substitutions using a similar methodology. While suggestive, this research is not conclusive because of two problems. First, the distinction between near and true synonyms raises the question of how to spot a synonym error if one occurs—is *ripe* truly a synonym of *done* or some other relationship? Second, speech errors were collected in both studies by a single expert collector using similar methods of validating and classifying errors. Prior research has shown that speech errors collected in the wild often present ambiguities as far as whether an error has occurred and how to analyze it (Alderete & Davies, 2019; Cutler, 1982; Ferber, 1995). We have no reason to doubt the assumptions of this prior work. But given their closely-aligned methods and reliance on a single data collector, we think independent support is necessary to answer Levelt’s question definitively.

Our aim here is to extend the empirical scope of this debate by investigating the errors in question in a large corpus of English speech errors. The design of our corpus allows a stronger test of Levelt’s question, with larger baselines, several measures for assessing error status, and methods that are more robust to known biases in speech error research. We give a detailed description of the distribution of synonym and subsumative errors within a larger analysis of the complete set of semantic errors. This larger analysis provides both an empirical basis for addressing Levelt’s question, as well as supporting further research on the nature and frequency of other types of semantic errors.

## 2. Methods

The speech error data were drawn from the Simon Fraser University Speech Error Database (SFUSED) English, a large database of roughly 10,000 speech errors collected from spontaneous English conversations (Alderete, 2019). A detailed account of its methods is given in Alderete and Davies (2019), but we summarize the key features here to contextualize the research. Errors were collected from high quality audio recordings of unscripted conversations (e.g., podcast series like *Astronomy Cast* and *Battleship Pretension*), rather than direct observation of spontaneous speech typical of older collections, in order to confirm the speech offline with replay and acoustic analysis. The errors were collected by a team composed of the first author and 18 linguistics undergraduate and graduate students who had undergone a month of training, which involved phonetic transcription, an introduction to psycholinguistics of speech errors, and listening tests in which collectors were given feedback on their error detection. The errors submitted by this team were then verified by an independent research analyst who checked that the submitted errors indeed met the description of valid speech errors (Dell, 1986) and then classified them using an established taxonomy of speech errors (Stemberger, 1993). The reliability and quality of data collected in this manner have been validated and shown to be less prone to the psychological and statistical biases that tend to distort speech error patterns (Alderete & Davies, 2019; Alderete & Tupper, 2018).

From this larger database, we extracted 1847 lexical substitution errors and 114 word blends (roughly 20% of the corpus) to probe the structure of lemma access failures. We then excluded certain lexical substitutions (e.g., errors with closed class items and errors with shared lemmas) to make them consistent with prior research (Harley & MacAndrew, 2001; Jaeger, 2005). The remaining 1094 lexical substitutions involved only nouns, adjectives, and verbs, and the vast majority (92.63%) did not change word class, respecting the well-known syntactic category constraint (Butterworth, 1982; Garrett, 1980). A data set containing these 1208 substitution and blend errors is available from the OSF page, https://osf.io/wruxf/, and includes longform entries with the complete linguistic context of the error and 21 categorical variables that can be used to cross-classify the data in the ways explained below. We hope that these detailed records can support further research beyond the scope of this project.

We developed a classification system based on the semantic relationships between error and intended words attested in prior work (Arnaud, 1999; Dell et al., 1997; Harley & MacAndrew, 2001; Jaeger, 2005). It includes co-hyponyms (or ‘coordinates’), antonyms, subsumatives, synonyms, thematically-related words, and semantically unrelated words. Two linguistics graduate students were recruited and trained to classify speech errors into these categories. In particular, student classifiers were given definitions of each category and several examples illustrating them, and then given a chance to ask questions about how to classify new errors until they felt confident about how to classify them. The speech errors to be classified were presented with their full linguistic context so that word meanings could be disambiguated by this context. To validate the analysis of each error, classifiers were also asked to state a super-ordinate category for each taxonomically related error and a theme for thematically related errors. Borderline cases could be assigned more than one relationship, but they were forced to pick the most appropriate analysis with these ambiguous cases. Agreement between classifications was moderate: 68.57% on the first choice and slightly higher between first or second (73.05%). Because agreement was moderate, a third analyst (first author) examined all of the errors and gave the best-out-of-three classification reported here.

Lexical substitutions can be semantically related, form-related (/oil/→*oral*), or both (/snails/→*snakes*), and past research has distinguished these classes to isolate conditioning effects (Fay & Cutler, 1977; Goldrick & Rapp, 2001). After experimenting with different measures of form-relatedness, we adapted the methods used in the Philadelphia Naming Test (Roach et al., 1996) to the task. Errors were deemed form-related if they had a non-trivial form resemblance: two shared segments in the same linear order in monosyllables (as in /phone/→*friend*, sharing sounds *f* and *n*) and at least three correctly ordered shared segments in polysyllabic words (e.g., /injury/→industry, sharing ɪ *n r* and *i*). With this measure, 20.30% of all lexical substitutions are form-related.

## 3. Results

Before probing the viability of synonym and subsumative errors, we flesh out some of the conditioning factors shaping the data. These factors are shown in sections bound by double lines in Table 1, which gives the relative frequencies of the six semantic categories by condition. Most lexical substitutions are noncontextual (80.44%) in the sense that the error word is not drawn from the linguistic context. This factor significantly affects the distribution of semantic categories (using traditional chi-square tests, χ^2^(5) = 45.98, *p* < 0.005): rates of thematic and semantically unrelated errors are far higher in contextual errors, and conversely, taxonomically related semantic errors are higher in noncontextual errors. This asymmetry is expected because mis-selections of words from context are less likely to be semantically related than words drawn from a cohort of semantic competitors in noncontextual errors.

**Table 1:** Relative frequencies of word errors by semantic category (columns) and contextuality, form-relatedness, correctedness, and error type (rows). Percentages in parentheses are by row totals, except for correctedness, which is by column total.


	CO-HYPONYMS	ANTONYMS	SYNONYMS	SUBSUMATIVES	THEMATIC	UNRELATED	TOTALS

Contextual	23 (10.75)	5 (2.34)	6 (2.80)	10 (4.67)	52 (24.30)	118 (55.14)	214

Noncontextual (NC)	222 (25.23)	41 (4.66)	77 (8.75)	61 (6.93)	153 (17.39)	326 (37.05)	880

Form-related	18 (7.73)	5 (2.15)	17 (7.30)	4 (1.72)	13 (5.58)	176 (75.54)	233

Not form-related (NFR)	227 (26.36)	41 (4.76)	66 (7.67)	67 (7.78)	192 (22.30)	268 (31.13)	861

Corrected	215 (87.76)	37 (80.43)	66 (79.52)	63 (88.73)	162 (79.02)	335 (75.45)	878

Not corrected	30 (12.24)	9 (19.57)	17 (20.48)	8 (11.27)	43 (20.98)	109 (24.55)	216

Word blends	23 (21.70)	4 (3.77)	38 (35.85)	5 (4.72)	15 (14.15)	21 (19.81)	106

Lexical substitutions	245 (22.39)	46 (4.20)	83 (7.59)	71 (6.49)	205 (18.74)	444 (40.59)	1094

NC and NFR	206 (30.70)	36 (5.37)	61 (9.09)	57 (8.49)	144 (21.46)	167 (24.89)	671


Form-relatedness also affects the counts by category (χ^2^(5) = 158.33, *p* < 0.001): non-form-related errors show much higher rates of semantic errors relative to unrelated errors, which again is expected if form-related words arise from phonological rather than semantic facilitation. Interestingly, 57 lexical substitutions (5.21%) are both semantically and form-related. Compared to the chance rates of these mixed errors from prior research (e.g., less than half of 1% in Dell et al., 1997), these errors are clearly above chance.

The relative frequencies by semantic category in lexical substitutions can also be compared to those of the 106 word blends in our corpus (down from 114 blends after removing eight blends sharing the same lemma, which were also excluded from lexical substitutions). The principal differences are that synonym errors are roughly five times more common in blends than lexical substitutions, which have corresponding drops in thematic and semantically unrelated errors.

As with prior research (Harley & MacAndrew, 2001; Jaeger, 2005), we focus on the noncontextual and non-form-related errors (Table 1, bottom row) because they are unaffected by linguistic context and form-encoding. The results line up with past reports arguing that both synonym and subsumative errors are viable lexical substitutions. The percentage occurrence for synonyms and subsumatives, 9% and 8%, respectively, compares with the rates found in Jaeger (2005): 6% and 9%, and Wan and Ting (2019): 13% and 10%. Thus, these errors are infrequent relative to co-hyponyms and thematic errors, but they are non-negligible and indeed more common than canonical word associates, antonyms.

To pursue specific hypotheses about how these errors arise, we provide a sample of the errors in Table 2. Though we found evidence of Jaeger’s claim that synonym errors arise from selectional restrictions, only 19.28% of the synonym errors were outright ungrammatical because of these constraints (Table 2A). More often, talkers erred with a synonym or a closely related word that is odd but still grammatical given its linguistic environment (Table 2B). However, these grammatical and sentence constraints are not the full story, because approximately half of these errors arise simply because the selected word does not exactly match the talker’s intentions, as in /dedicated/→*devoted* from Table 2C.

**Table 2:** Synonym and subsumative errors (with /intended/→error, SFUSED-English record ID number, and podcast source).


A. Synonyms: Same concept, ungrammatical by selectional restrictions: 16 (19.28%) What did we /say my, what did we tell my parents we’re seeing? (/tell/→say, 2783, *Battleship Pretension*) I can see how one is totally /badder, worse than the other. (/worse/→badder, 205, *Go Bayside*) We can only see a /few percentage of the whole universe. (/small/→few, 2358, *Astronomy Cast*)

B. Synonyms: Same/close concept, but odd: 26 (31.33%) Don’t bring those kids in the /home, house. (/house/→home, 9543, *This Feels Terrible*) Professional /level, uh professional quality ear buds, yes. (/quality/→level, 6688, *Battleship Pretension*) I didn’t know this at the /point, time that they were dating. (/time/→point, 8337, *This Feels Terrible)*

C. Synonyms: same/close concept, but not right: 41 (49.40%) And if we don’t understand gravity at those /long [d]= large distances then. (/large/→ long, 4593, *Astronomy Cast*) We’ve /dedicated, not dedicated, devoted a whole episode to him. (/devoted/→dedicated, 4557, *Battleship Pretension*) The /Dark Ages, the Middle Ages were stupid. (/Middle Ages/→Dark Ages, 3224, *We Have Concerns*)

D. Subsumatives: super-ordinates 47 (66.20%) So maybe it was too nitrogen rich and wasn’t putting out /plants, er, not plants, flowers. (/flowers/→plants, 1739, direct observation) … about the feelings of the /people that she, the men that she loves (/men/→people, 5238, Battleship Pretension) If there’s one thing to with /anger, rage that’s healthy, it’s to dance it out. (/rage/→anger, 5361, *We Have Concerns*)

E. Subsumatives: subordinates: 24 (33.80%) Is there anything else you wanted to say about that /song, or that, that music? (/music/→song, 7248, *Battleship Pretension*) You have a complex /water cycle, or liquid cycle rather. (/liquid/→water, 9906, *Astronomy Cast*) I like melted /congee. (/rice/→congee, 4359, direct observation)


Talkers often correct their errors, and such self-corrections are a form of evidence that an error has occurred. While Jaeger (2005: 343) remarks subsumatives tend to be corrected to convey the correct level of specificity, we did not find evidence that the categories in question differ in correctedness (Table 1). The majority of lexical substitutions are corrected, regardless of semantic category, and while subsumatives have a slightly higher rate, they compare with the rate of correction in co-hyponym errors. However, subsumatives are disproportionally super-ordinates (66.20%) relative to sub-ordinates (see Table 2 D/E), perhaps revealing a bias for the more general category.

Determining if an utterance in the wild contains a true speech error can involve subtle judgements with synonyms and subsumatives, leaving doubt in error status in some cases. Our data, however, leaves no doubt about the existence of these error types. First, roughly half of all synonym errors arise from selectional restrictions or general sentence constraint. Such constraints are commonly used as diagnostics for lexical and morpho-syntactic errors (Jaeger, 2005; Stemberger, 1982/1985), so these must be registered as errors. Second, synonym and subsumative errors are corrected at rates comparable to other lexical substitution errors. Third, we have examined the relative rate of occurrence of the six semantic categories detected by nine individual data collectors to determine if the errors in question are broadly recognized as errors. The data from these data nine collectors were a subset of the larger corpus (*n* = 819, Table 3) because errors from some data collectors with small overall counts of lexical substitution errors (i.e., less than 14) were removed to ensure robust frequency distributions across the six semantic categories. In this data set, all but one collector detected nonzero counts of both synonyms and subsumatives, and the two zero counts (for antonyms and synonyms) came from a collector with low overall counts (*n* = 21), suggesting the zeros are a baseline effect. In sum, word substitutions involving synonyms and subsumatives were judged by several independent data collectors as errors, without any explicit instructions to look for errors of these types.

**Table 3:** Total counts by category (percentage occurrence), mean detection rates (i.e., counts/hours of listening), and 95% confidence interval for estimated Poisson rates.


	CO-HYPONYMS	ANTONYMS	SYNONYMS	SUBSUMATIVES	THEMATIC	UNRELATED

Total (%)	194 (23.69)	34 (4.15)	70 (8.55)	57 (6.96)	158 (19.29)	306 (37.36)

Detection rates	0.83	0.12	0.27	0.22	0.47	1.09

CI 95% Poisson	221.30, 166.70	47.51, 23.55	88.44, 54.57	73.85, 43.17	182.64, 133.36	340.29, 271.71


**Figure 1:**
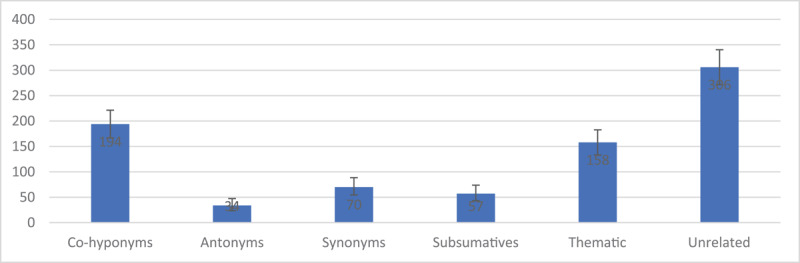
Estimates of Poisson Rates with 95% CI.

This subset of 819 fully independent lexical substitution errors can be summed and used to establish a stronger statistical basis for investigating all six semantic categories. Assuming that these summed frequencies have a Poisson distribution (since the sum of a Poisson random variable is still a Poisson), we estimated the population means and 95% confidence intervals using the Matlab function poissfit (Figure 1 and Table 3). This analysis results in three frequency classes with non-overlapping confidence intervals: High (unrelated), Mid (co-hyponyms, thematic), and Low (antonyms, synonyms, and subsumatives). These classes are correlated with the mean detection rates across data collectors, though the correlation is weak in some comparisons. For example, there are 112 more semantically unrelated errors than co-hyponym errors, a 58% boost of the latter. This is consistent with an increase in mean detection rates from 0.83 (or about five errors every six hours) to 1.09 (roughly 6.5 errors every six hours), though this is only a 31% increase in detection.

These results support our general contention that synonym and subsumative errors are non-negligible. Indeed, synonyms are twice as frequent as canonical lexical substitutions, antonyms (and they have non-overlapping confidence intervals). We hope that this quantitative analysis and the associated data set will support future investigation of these semantic categories more broadly.

## 4. Discussion

We have investigated the distribution of semantic categories in lexical substitution errors in spontaneous speech to assess the viability of errors with synonyms, super-ordinates, and sub-ordinates and to give a general account of the frequency distributions of lexical substitution and word blend errors. After removing the effects of linguistic context and form-encoding, these categories had relatively low, but non-negligible frequencies, supporting Jaeger (2005). It could be that synonym and subsumatives are actually more frequent in natural speech and just spotted less frequently by our listeners. However, they are not flagged as errors with speaker self-correction more than other categories, so we believe that the attested counts given here are not too far off from the true distribution.

As for model implications, the existence of synonym and subsumative errors does not pose a problem because current models of lemma selection predict them. The problem is, perhaps paradoxically, how to inhibit their occurrence. If activation from shared features or concepts can lead to mis-selections of associated words, then word pairs that share more of this structure should have still more mis-selections. In taxonomically related word pairs, co-hyponym errors are roughly three times more common than synonym and subsumative errors. One proposal is that these differences arise not from underlying processing, but instead reflect constraints in the lexicon (Jaeger, 2005). For example, perhaps the English lexicon has more co-hyponyms than synonyms, and so there are many more opportunities for errors with this type. This problem resembles the account of mixed errors, which, like synonyms, have more sources of activation (both semantic and formal), but occur at lower rates than semantic errors (Dell et al., 1997). This approach also has relevance to the overlooked problem of semantically unrelated errors, which are the most frequent semantic category overall. Unrelated lemmas may produce little competition, but as the complement class in the entire lexicon, they provide far more opportunity for mis-selection than any other category.

However, if error opportunity does not give a complete account of the distributional differences, then the mechanisms of lemma selection itself can be adapted to these new facts. We note that conceptual links in WEAVER++ are labelled pointers designed to distinguish relationships between concepts (Roelofs, 1992) and may, through weighting, be up to the task of inhibiting activation flow between certain concepts.

Our findings also raise interesting empirical issues about how semantic relationships are characterized. Classifying speech errors into the six semantic categories is a challenge for human analysts, as shown by moderate agreement in classification, as well as the need for multiple classifications of the same error and near synonyms as a category as opposed to true synonyms. These problems resemble the limits of meaning construal with discrete semantic attributes that have led many researchers to use computational methods for assessing word to word relationships (Vinson et al., 2014). For example, distributional models have developed explicit methods of quantifying semantic similarity, like cosine similarity, that draw on corpora instead of human intuitions. The continuous measures produced by such analyses can be used to validate the classification from human researchers, and at the same time, they open up new analyses for speech error data that allow for grades of semantic relatedness that classes like near synonyms seem to require.

## Data Accessibility Statement

All of the data reported here can be accessed at the Open Science Foundation project page https://osf.io/wruxf/. The data on this repository includes the longform entries of each speech error with the complete linguistic context and 21 categorical variables for cross-classifying the data.
